# Loss of GPR109A/HCAR2 induces aging-associated hepatic steatosis

**DOI:** 10.18632/aging.101743

**Published:** 2019-01-18

**Authors:** Ravirajsinh N. Jadeja, Malita A. Jones, Ollya Fromal, Folami L. Powell, Sandeep Khurana, Nagendra Singh, Pamela M. Martin

**Affiliations:** 1 Department of Biochemistry and Molecular Biology, Medical College of Georgia at Augusta University, Augusta, GA 30912, USA; 2 James and Jean Culver Vision Discovery Institute, Medical College of Georgia at Augusta University, Augusta, GA 30912, USA; 3 Education Innovation Institute, Medical College of Georgia at Augusta University, Augusta, GA 30912, USA; 4 Division of Gastroenterology, Hepatology and Nutrition and Weight Management, Geisinger Medical Center, Danville, PA 17822, USA; 5 Georgia Cancer Center, Medical College of Georgia at Augusta University, Augusta, GA 30912, USA; 6 Department of Ophthalmology, Medical College of Georgia at Augusta University, Augusta, GA 30912, USA

**Keywords:** aging, steatosis, GPR109A, lipid metabolism

## Abstract

GPR109A agonists have been used for the treatment of obesity however, the role of GPR109A in regulating aging-associated alterations in lipid metabolism is unknown. In this study we used *Gpr109a^-/-^* mice to investigate the effect of aging in the regulation of lipid accumulation. We observed that in mouse and human livers, in addition to Kupffer cells, GPR109A is expressed in hepatocytes. Over 12 months, compared to wild type (WT), *Gpr109a^-/-^* mice gained significantly more weight. Food intake and levels of serum lipids were similar among both groups. Compared to age-matched WT mice, 12-months old *Gpr109a^-/-^* mice had significantly increased liver weight, hepatic steatosis and serum markers of liver injury. The fatty liver phenotype in *Gpr109a^-/-^* mice was associated with increased hepatic expression of lipogenesis genes and decreased expression of lipolysis genes. *Gpr109a^-/-^* mice had significantly increased fat tissues, which was associated with significant increase in adipocyte diameter and surface area. Adipose tissue from *Gpr109a^-/-^* mice had increased expression of lipogenesis genes; however, expression of lipolytic genes was similar in both groups. Collectively, these results indicate that during aging, GPR109A modulates *de novo* lipid accumulation in liver and adipose tissue, and its dysregulation can lead to age-associated obesity and hepatic steatosis.

## Introduction

Aging is a process of multidimensional organism decline. By 2050, 22% of the world’s population will be greater than 60 years of age [1]. Aging itself is not a disease however, it is one of the leading risk factors for many debilitating diseases [2,3]. This is not surprising especially given that the liver, a major regulator of metabolic function, is prominently impacted in the aging process. The age-associated alteration of liver structure and function is supported by histologic findings such as hepatocyte enlargement, the presence of an increased number of binucleated cells, and reduced mitochondrial density that present with increased age even in the absence of disease [4,5]. Further, experimental and human clinical data demonstrate convincingly a correlative relationship between risk of development, advanced progression and mortality from common disorders of the liver such as non-alcoholic steatohepatitis (NASH), hepatic fibrosis and hepatocellular carcinoma (HCC) and age [6,7]. Importantly, the impact of age-associated liver damage or dysfunction is broad. Alterations in liver structure and function are linked causally to the development and/or progression of metabolic diseases such as diabetes, cardiovascular disease, cancer and others [8]. Thus, understating better mechanisms that underlie age-related liver damage and/or dysfunction such that strategies to protect and preserve this vital metabolic organ can be developed is of paramount importance. This age-associated risk is further magnified in conjunction with obesity. A number of mechanisms such as increased dietary lipid consumption and hepatic lipid synthesis or decreased lipid catabolism [6–8] have been evaluated to explain the impact of aging on the aforementioned conditions however, several important questions remained unanswered (i.e., is the higher prevalence of NAFLD seen in the elderly population a result of physiological changes related to aging or is it a reflection of lifestyle-associated factors (obesity and increased calorie intake)? Is aging then an actual risk factor for liver diseases, or a merely a bystander [7]?). With a goal of improving the current understanding of these topics, in the present study we focused on the G-protein coupled receptor GPR109A.

GPR109A, also known as hydroxycarboxylic acid receptor 2 (HCAR2), HM74A or PUMA-G, is located on chromosome 12q24.3. GPR109A expression has been demonstrated in a variety of cells and tissue types including adipocytes of white and brown adipose tissue, keratinocytes and immune cells, and epithelial cells of the colon and retina [9–15]. The receptor has been characterized as a metabolic sensor, modulating cell signaling that is coupled to energy and lipid metabolism as well as immune cell function both directly and indirectly when activated in response to changes in metabolic and/or immune status [16]. Despite the essential role of the liver in maintenance of metabolic homeostasis and the related key functional role of GPR109A-mediated signaling in this process, GPR109A expression has not been evaluated thoroughly in liver. In fact, some controversy exists regarding whether or not the receptor is expressed in this tissue. Therefore, in the present study we examined GPR109A expression in normal human and mouse livers and, evaluated the impact of receptor expression on age-associated steatosis, a condition that commonly precedes and/or contributes to serious functional abnormality and disease development and progression in liver. Specifically, using wild type and *Gpr109a^-/-^* mice we confirmed GPR109A expression in human and mouse liver and demonstrated an age-dependent decline in its expression in this tissue. Loss of GPR109A expression was associated with increased visceral and hepatic fat accumulation, a phenotype that our subsequent molecular studies indicated to be due largely to the increased expression of lipogenic enzymes in liver and adipose tissues.

## RESULTS

### Loss of GPR109A induced significant weight gain without affecting food intake

**Figure 1:**
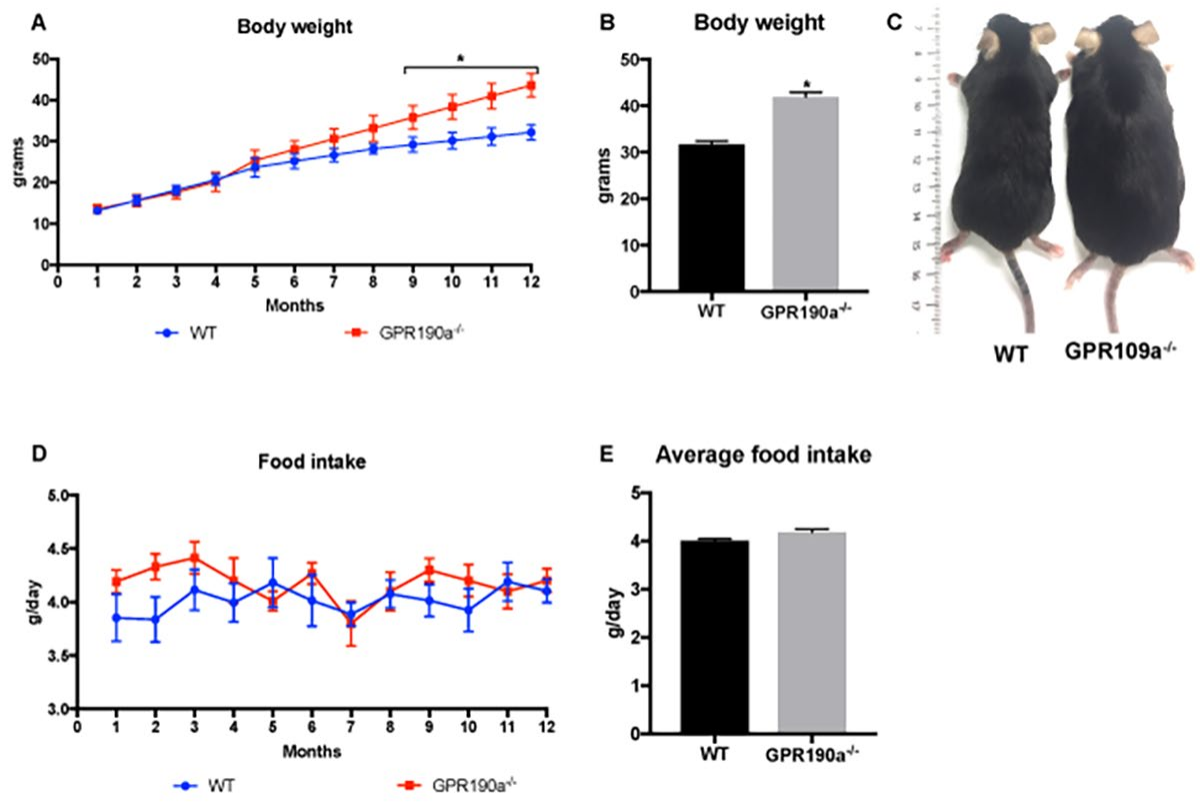
**Loss of GPR109A induced significant weight gain without affecting food intake. (A)** Body weight of WT and *Gpr109a^-/-^* mice was recorded weekly for a period of 12 months and **(B)** final body weight at the end of 12 months was calculated. **(C)** Visual appearance from a dorsal view of 12-month-old WT and *Gpr109a^-/-^* mouse shows significant differences in the body weight. **(D)** Food intake of WT and *Gpr109a^-/-^* mice was recorded weekly for a period of 12 months and **(E)** average food intake was calculated. Data are presented as mean ± S.E.M for (n=6). *p<0.05 vs. WT.

Male, wild type (*Gpr109a^+/+^,* WT) and *Gpr109a^−/−^* (knockout, KO) mice were maintained in our laboratory on a diet of standard rodent chow for 12 months. Body weight and food intake were monitored continuously. By 20 weeks of age, KO mice were noticeably larger in body mass than their age-matched WT counterparts. This is supported by body weight measurements that revealed that by 9 months of age, *Gpr109a^-/-^* mice weighed significantly more than age-matched WT mice (Fig. 1A-C) despite the fact that food intake did not differ significantly between the two groups (Fig. 1D-E).

### Increased weight gain in *Gpr109a^-/-^* mice is not associated with change in circulating lipids

To determine whether the increase in weight in knockout mice is linked to alterations in circulating lipid levels, we next evaluated circulating lipid profiles in *Gpr109a^-/^*^-^ and age-matched WT mice. Surprisingly, circulating lipid levels in *Gpr109a^-/-^* mice were similar to 12-month-old WT mice (Fig. S1A-E). Therefore, we concluded that circulating lipids do not contribute towards the obese phenotype in aged *Gpr109a^-/-^* mice. Alternately, we speculated that GPR109A may modulate lipid metabolism in adipocytes and liver. GPR109A expression has been confirmed in adipose tissue [17]. However, in order for this to speculation to be valid, the receptor must also be expressed in liver.

### GPR109A is expressed in human and mouse livers

**Figure 2:**
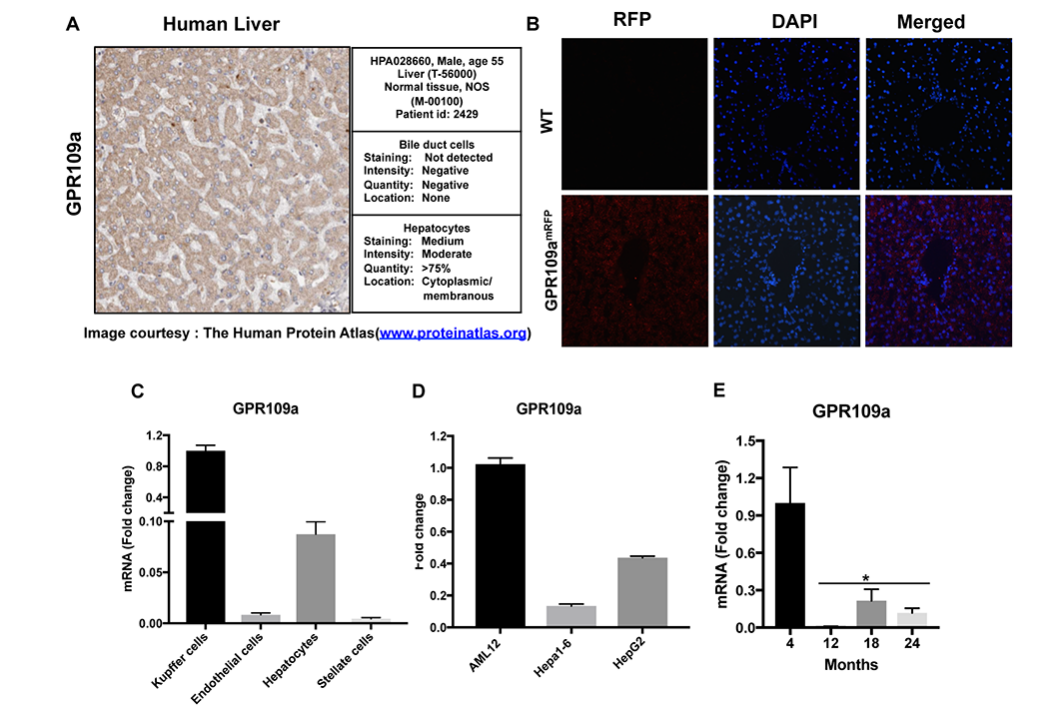
**GPR109A is expressed in Hepatocytes. (A)** An image of human liver section obtained from the Human protein atlas (www.proteinatlas.org) shows moderate immunoreactivity for GPR109A in hepatocytes. **(B)** Immunofluorescence staining of liver sections from WT and *Gpr109a^mRFP^* mice using anti-RFPtag antibody confirm receptor expression in liver. **(C)** GPR109A mRNA expression in different cell populations isolated from mouse liver was evaluated by qPCR assay. **(D)** GPR109A mRNA expression in different hepatocyte cell lines of mouse and human origin was evaluated by qPCR assay. **(E)** GPR109A mRNA expression in liver of 4, 12, 18 and 24-month-old WT mice. Data are presented as mean ± S.E.M for (n=3-6). *p<0.05 vs. 4-month old mice.

There have been mixed reports regarding the expression of GPR109A in liver. Some studies have reported that it is not expressed in liver whereas others report that it is indeed expressed but, only at very low levels. To obtain a definitive answer, we performed in-depth characterization of GPR109A expression in liver sections and in isolated liver cell populations. As shown in Figure 2A, GPR109A expression data obtained from The Human Protein Atlas [18] shows moderate staining reactivity in normal human liver. Using *Gpr109a^mRFP^* mice, mice that contain red fluorescence protein (RFP)-tagged to the GPR109A promoter [13], we confirmed this finding in cryosectioned mouse livers. Red fluorescence, indicative of RFP positivity, was detected in liver sections prepared from *Gpr109a^mRFP^* mice (Fig. 2B). Seventy to eighty percent of the liver is made up of hepatocytes however, additional cell types are present. Therefore, to determine which specific cell types within liver express GPR109A is expressed, we monitored GPR109A mRNA expression in different liver cell populations that were isolated from normal mouse livers per our established protocol [19]. Consistent with reports by others, hepatocyte-specific GPR109A expression was detected. Regarding expression of the receptor in additional cell types, mRNA transcripts specific to GPR109A were additionally detected in Kupffer cells, endothelial and stellate cells. GPR109A expression was highest in Kupffer cells, lowest in endothelial and stellate cells, and comparatively intermediates in hepatocytes (Fig. 2C). This was confirmed by our additional analyses of GPR109A expression in established mouse and human hepatocyte cell lines in which we found the receptor to be expressed at variable levels in each (Fig.2D). We additionally evaluated GPR109A mRNA expression in liver samples obtained from 4, 12, 18 and 24-month-old wild-type C57BL/6J mice. Collectively, these data indicate that GPR109A is expressed in the liver and that its expression decreases significantly in aging mice (Fig. 2E).

### Absence of GPR109A induces age-associated hepatic steatosis in mice

**Figure 3:**
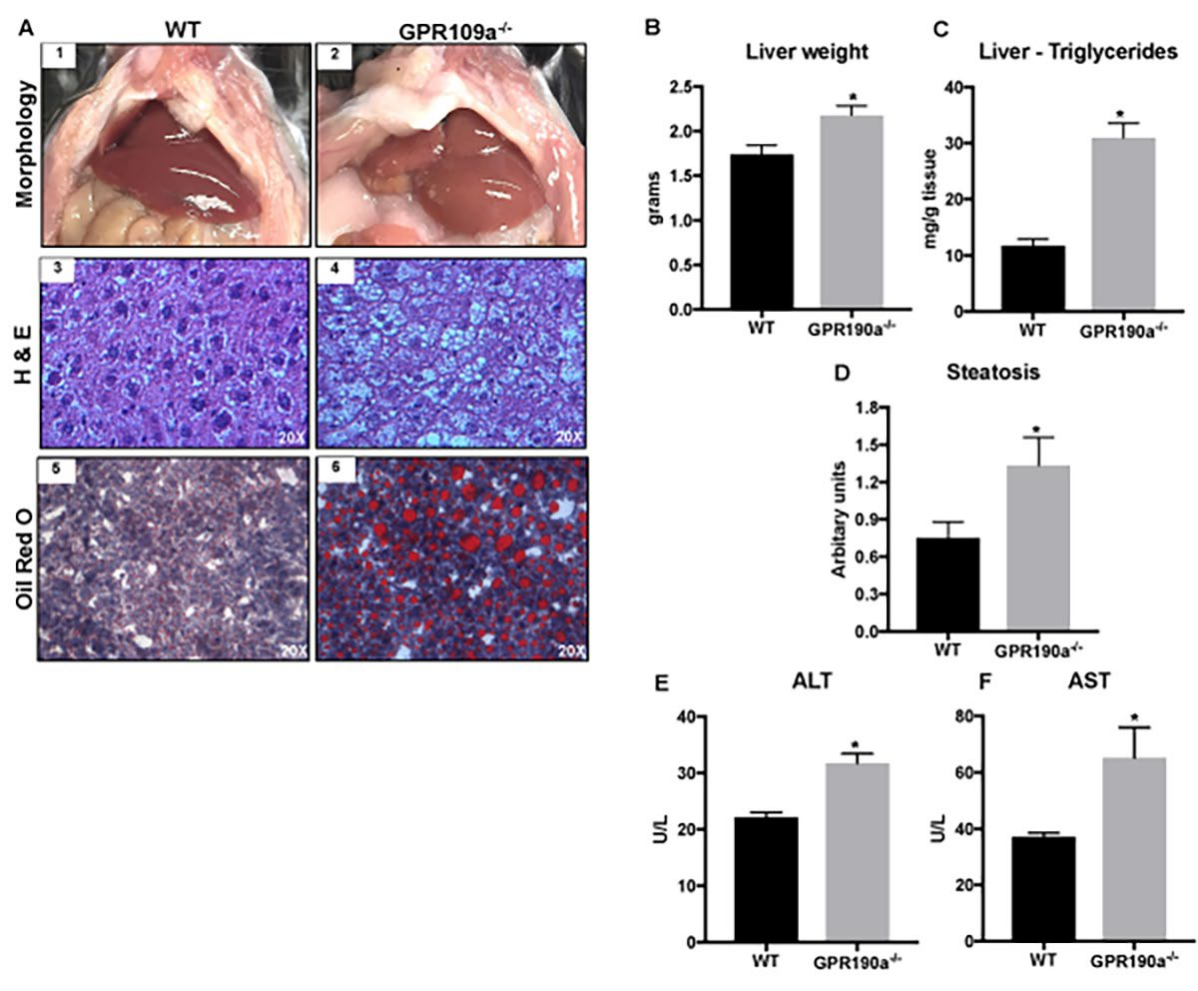
**Absence of GPR109A induces age-associated hepatic steatosis in mice. (A)** Morphological appearance (1-2), hematoxylin and eosin (3-4) and Oil red O staining of 12 month old WT and *Gpr109a^-/-^* mouse liver sections (20X) (5-6). **(B)** Changes in liver weight and **(C)** triglyceride content of 12-month-old WT and *Gpr109a^-/-^* mice. **(D)** Semi-quantitative scoring system was used to assess hepatocyte steatosis (0, 1-5% of total area; 1, 5 – 33% of the total area; 2, 33 – 66% of the total area; 3, >66% of the total area; results represented in arbitrary units. **(E-F)** Changes in the circulating levels of liver injury markers. Data is represented as mean ± S.E.M for (n=6). *p<0.05 vs. WT.

We have now confirmed that aging GPR109A knockout mice gain significantly more weight than WT mice of comparable sex and age, a factor not linked to differences in dietary intake or circulating lipids. As such, we hypothesized that the difference in weight between the two groups stems from differences in the metabolism of lipids and in turn, fat accumulation related directly to the presence or absence of GPR109A expression in liver and adipose tissue, the tissues principally responsible for maintaining lipid homeostasis in mammals. To validate this hypothesis, we looked first to the liver, a tissue that we now know definitively expresses GPR109A. Gross morphological examination of the abdominal cavity of WT and *Gpr109a^-/-^* mice at 12 months of age, an age at which highly significant differences in weight gain were detected, revealed the presence of substantially more visceral fat and liver adiposity, as evidenced by livers that were consistently enlarged and pale compared to WT counterparts. As shown in Fig. 3A, morphological assessment of hematoxylin and eosin-stained sections of livers from *Gpr109a^-/-^* mice confirmed a liver phenotype consistent with steatosis. To follow-up on these observations liver weights were obtained and Oil Red O and hepatocyte triglyceride (TG) assays were performed. Not only were liver weights in *Gpr109a^-/-^* mice significantly greater than in WT (Fig. 3B) but, Oil Red O staining and hepatocyte triglyceride (TG) assays additionally confirmed the excessive accumulation of fat in *Gpr109a^-/-^* mice compared to WT (Fig.3A & C). These data were further supported by semi-quantitative scoring, which indicated hepatic steatosis in *Gpr109a^-/-^* mice (Fig. 3D). As such, we next evaluated levels of aspartate aminotransferase (AST) and alanine aminotransferase (ALT), circulating markers of hepatocyte injury. ALT and AST levels were significantly higher in *Gpr109a^-/-^* mice than in age-matched WT controls (Fig. 3E-F).

### Increased lipogenesis and decreased lipolysis accounts for hepatocyte fat accumulation in *Gpr109a^-/-^* mice

**Figure 4:**
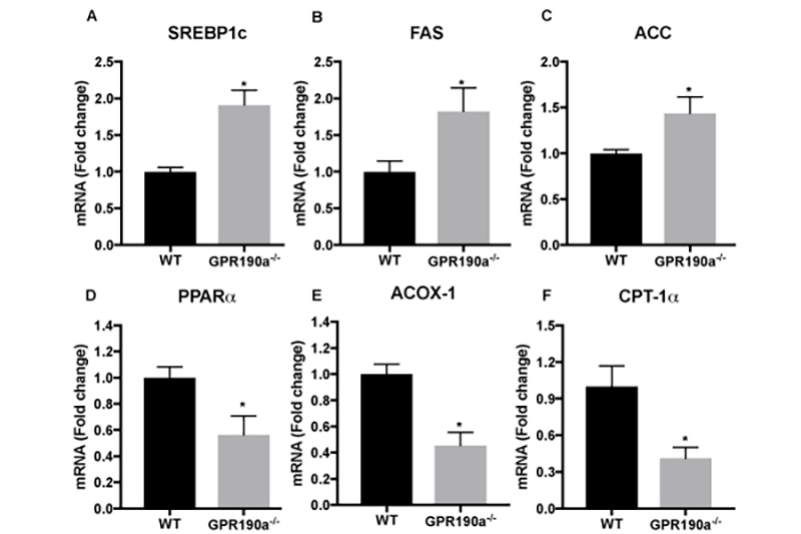
**Increased lipogenesis and decreased lipolysis accounts for hepatocyte fat accumulation in *Gpr109a^-/-^* mice. (A-F)** mRNA expression of genes regulating hepatocyte lipid metabolism was performed by qPCR assay. Data are presented as mean ± S.E.M for (n=4). *p<0.05 vs. WT. SREBP1c; sterol regulatory element-binding transcription factor 1, FAS; fatty acid synthase, ACC; acetyl-CoA carboxylase, PPARα; peroxisome proliferator-activated receptor alpha, ACOX-1; Acyl-CoA Oxidase 1, CPT1A; Carnitine palmitoyltransferase IA.

Hepatocyte fat content is maintained by a fine-tuned balance between lipogenesis and lipolysis. As such, we evaluated the expression of key genes involved in both pathways using qPCR and gene-specific primers (Table 1). The expression of lipogenic genes including the transcription factor SREBP1c (sterol regulatory element-binding protein 1c) and enzymes, FAS (fatty acid synthase) and ACC (acetyl-CoA carboxylase) were upregulated significantly in *Gpr109a^-/-^* mouse liver (Fig. 4A-C). Alternately, lipolytic genes such as the transcription factor PPARα (peroxisome proliferator-activated receptor alpha) and related enzymes, ACOX1 (acyl-CoA oxidase) and CPT-1(carnitine palmitoyltransferase 1), were significantly downregulated (Fig. 4D-F).

**Table 1:** Primer sequences used for qPCR.

**Gene**	**Forward (5’-3’)**	**Reverse (5’-3’)**
***Homo sapiens***		
GPR109a	GGACAACTATGTGAGGCGTTGG	GGGCTGGAGAAGTAGTACACC
18S	CCCGTTGAACCCCATTCGT	GCCTCACTAAACCATCCAATCGGTA
***Mus musculus***		
Acc	TCCCCTGCCAGCAGATAG	TGAAGAAGACCTCTCGGTCC
Acox1	CTCACTCGAAGCCAGCGTTA	CGGTGCACAGAGTTTTAAACCA
Atgl	TTAGGAGGAATGCCCTGCTG	CTGCTCTTTCATCCACCGGATA
Cebp1	GATTCCTGCTTCCTCTCGGG	TCCCCAACACCTAAGTCCCT
Cpt-1	CTCCGCTCGCTCATTCCG	TGCCATTCTTGAATCGGATGAACTT
Fas	TGAAGAAGACCTCTCGGTCC	CATAGAGCCCAGCCTTCCAT
Gpr109a	GTTACAACTTCAGGTGGCACGAT	CTCCACACTAGTGCTTCGGTTATT
Hsl	GCTGGATCTGCACTCTACCA	TTTTCCCTTTCGCAGCAACT
Pparα	GCAGCCTCAGCCAACTTGAAG	CGAACTTGACCAGCCACAAAC
Pparλ	ATGGGTGAAACTCTGGGAGAT	ATGGTAATTTCTTGTGAAGTGCTCA
Scd1	AGAGAACTGGAGACGGGAGT	AACACCCCGATAGCAATATCCA
Srebp1c	CCCGGCTATTCCGTGAACAT	AGAACTCCCTGTCTCCGTCA
18s	CCAGAGCGAAAGCATTTGCCAAGA	AGCATGCCAGAGTCTCGTTCGTTA

### Aging leads to adipocyte hypertrophy in *Gpr109a^-/-^* mice

**Figure 5:**
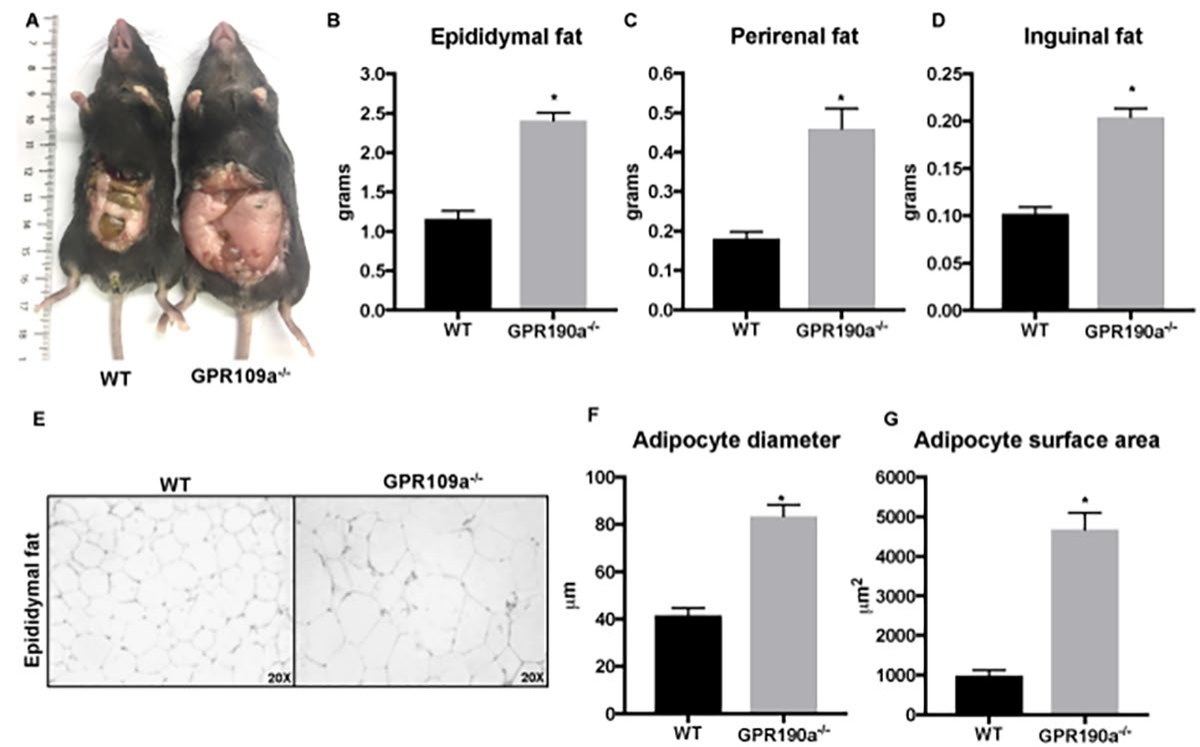
**Age-associated adipocyte hypertrophy is a feature of *Gpr109a^-/-^* mice. (A-D)** 12-month-old *Gpr109a^-/-^* mice had more visceral fat accumulation compared to age-matched WT mice. **(E)** Representative images (20X magnification) of hematoxylin and eosin-staining and **(F-G)** histo-morphometric evaluations performed using cross-sections of epididymal fat pads isolated from 12-month old WT and *Gpr109a^-/-^* mice. Data is represented as mean ± S.E.M for (n=4). *p<0.05 vs. WT.

Because notable differences were detected also in visceral fat accumulation in GPR109A knockout mice compared to WT mice (Fig. 5A-D), we performed additional histologic studies of adipose tissue. Evaluation of hematoxylin and eosin-stained sections of epididymal fat pad revealed increased adipocyte diameter and surface area (quantified using Adiposoft-Image J), indicating significant hypertrophy of adipocytes in *Gpr109a^-/-^* mice compared to WT mice (Fig. 5E-G).

### Aging-associated fat accumulation in *Gpr109a^-/-^* mice is via increased lipogenesis in adipose tissue

**Figure 6:**
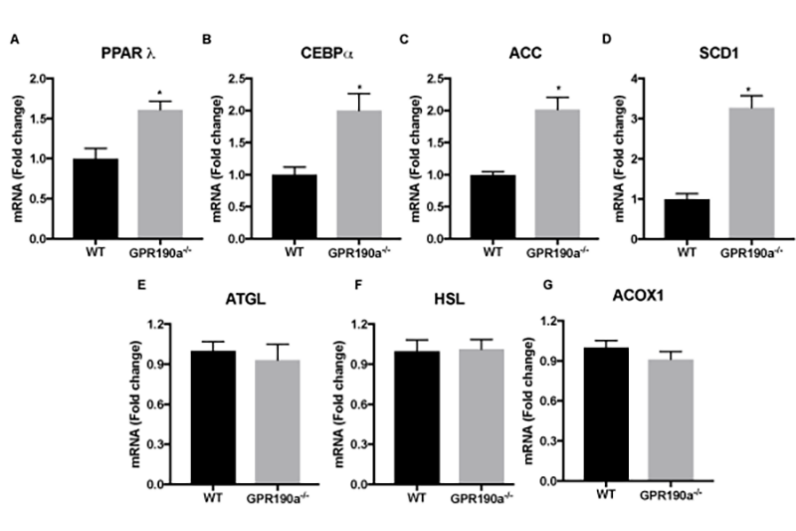
**Age-associated fat accumulates in *Gpr109a^-/-^* mice via increased lipogenesis. (A-G)** mRNA expression of genes regulating hepatocyte lipid metabolism was performed by qPCR assay. Data is represented as mean ± S.E.M for (n=4). *p<0.05 vs. WT. PPAR-γ; peroxisome proliferator-activated receptor gamma, CEBPα; CCAAT/enhancer-binding protein alpha, ACC; acetyl-CoA carboxylase, SCD-1; stearoyl-CoA desaturase-1, ATGL; adipose triglyceride lipase, HSL; hormone-sensitive lipase, ACOX-1; Acyl-CoA Oxidase 1.

To determine whether the hypertrophy of adipocytes in knockout mice is related to alterations in the expression of lipogenic and/or lipolytic genes, we examined key genes in each pathway just as we did in our studies of liver tissue. In accordance with our observations in liver tissue, expression of the key transcription factors (PPAR-γ; peroxisome proliferator-activated receptor gamma and CEBPα; CCAAT/enhancer-binding protein alpha) and enzymes controlling lipogenesis (ACC; acetyl-CoA carboxylase, SCD-1; stearoyl-CoA desaturase-1) were significantly up regulated in 12-month-old *Gpr109a^-/-^* mice compared to age-matched WT mice (Fig.6 A-D). However, contrary to our findings in liver, the expression of enzymes controlling lipolysis (ATGL; adipose triglyceride lipase, HSL; hormone-sensitive lipase, ACOX-1; Acyl-CoA Oxidase 1) were not significantly altered (Fig.6 E-G). This suggests that the absence of GPR109A expression in liver and adipose tissue induces differential metabolic regulation to enhance fat accumulation in each of these tissues.

## DISCUSSION

Aging and obesity are major risk factors for development and progression of metabolic diseases such as diabetes, cardiovascular disease and cancer [20]. The liver, a central regulator of metabolism, is impacted greatly in aging and, dysfunction of or damage to this organ in age- and non-age-related conditions is associated directly with obesity [21,22]. Thus, whether aging and obesity are viewed collectively or independently, the involvement of the liver in these conditions cannot be overlooked. Non-alcoholic fatty liver disease (NAFLD) is the most common liver disease in the world [20]. The strong association between aging, fat accumulation and liver dysfunction is exemplified by the increased risk of development of hepatic steatosis, its progression to and mortality from NALFD in the aging population [2,5]. Additionally, increases in the generation of pro-oxidant stimuli and related inflammation, factors that can be brought on by any number of circumstances but interestingly are quite commonly elevated in aging, contribute to the progression of NAFLD to non-alcoholic steatohepatitis (NASH), cirrhosis and HCC [23,24]. Experimental studies and human clinical data have revealed Increases in *de novo* lipogenesis coupled with impaired fatty acid oxidation in liver as mechanisms to explain the enhanced development and progression of the aforementioned liver complications in aging [25] however, the specific mechanisms that regulate these processes at the gene level are not well understood. With this is mind, we focused on GPR109A.

GPR109A was first identified as the receptor responsible for the pharmacological actions of niacin, a lipid-lowering drug in clinical use for more than 40 years [9–11]. Beta-hydroxybutyrate, identified presently as the only endogenous agonist of the receptor, has too been demonstrated to have some lipid lowering effects [26]. Interestingly, just as some controversy exists regarding whether GPR109A is expressed in liver or not, reports as to whether the lipid-lowering effects of niacin and beta-hydroxybutyrate stem principally from their interaction with the receptor are too conflicting [27–30]. However, having maintained our own colony of GPR109A knockout mice for several years now, there are several observations that we find to be undeniably consistent. *Gpr109a*-null mice become significantly obese in comparison to age and gender matched C57BL/6J mice. This physical characteristic is readily observable upon side-by-side comparison of wild type and knockout animals in normal housing conditions and more robustly apparent upon gross surgical examination of the abdominal cavity. Indeed, a liver phenotype consistent with hepatic steatosis (livers that were significantly enlarged in size and pale and splotchy in color) is a common finding. Fibrosis and nodules are additionally detected commonly in the livers of knockout mice. Alternately, livers of wild-type mice remain comparably normal in size and appearance even in advanced age. These observations suggest that GPR109A may have some regulatory impact on lipid metabolism and related fat accumulation both in adipose tissue, a tissue previously confirmed to express GPR109A and, in liver, a tissue essential also to lipid metabolism but one in which until now, expression of the receptor was uncertain. This prompted the present study in which we (a) thoroughly and systematically monitored body weight, food intake, the circulating lipid profile and morphology of liver and adipose tissues in age and gender matched wild type and GPR109A knockout mice and, (b) demonstrated convincingly the liver-specific expression of GPR109A and the significance of its expression with respect to the expression of key regulatory genes in lipid metabolism.

Consistent with our raw visual observations, quantifiably significant differences in weight gain and related fat accumulation were detected in GPR109A knockout mice compared to age and gender matched wild-type mice despite similarities in food intake between the two groups. Morphological and histological analyses of adipose tissue revealed an inverse relationship between the expression of key lipogenic genes, adipocyte hypertrophy and GPR109A expression. This explained in part the significant difference in visceral fat accumulation in aged GPR109A knockout mice compared to wild-type mice but did not account fully for the fatty liver phenotype, especially since there have been conflicting reports of GPR109A expression in hepatocytes [12,16,24,25]. To address this issue, we carried out a detailed characterization of GPR109A expression in cryosections sections of liver and in isolated liver cell types and, cross-referenced our laboratory findings with data available in the human protein atlas, a Swedish-based program that maps all the human proteins in cells, tissues and organs [18]. Because we did not have ready access to normal human liver specimen, we searched the human protein atlas for information relevant to GPR109A. Interestingly, the database included characterization data on the expression of GPR109A in human liver [18]. This was supported further by our analyses of *Gpr109a^mRFP^* mouse livers and isolated liver cell populations. Based upon these fluorescence histochemical studies and related molecular analyses was clear that GPR109A was expressed in normal hepatocyes. This we confirmed at the molecular level in murine primary hepatocytes and in several well-established human hepatocyte cell lines. We additionally detected GPR109A expression in other liver cell types. The expression of the receptor was highest in Kupffer cells. This was not completely surprising given that Kupffer cells serve as resident macrophages within liver and GPR109A is known to be expressed robustly in macrophages as reflected by the name that was ascribed to the receptor upon its initial discovery, PUMA-G, which means “protein upregulated in macrophages by interferon-gamma”. Kupffer cells line the liver sinusoids to participate in immune surveillance, secreting soluble factors upon activation that regulate their own phenotype and function as well as that of hepatocytes and other neighboring cells [31]. Importantly, Kupffer cells have been demonstrated to be pivotal players in lipid metabolism, immune regulation and therefore to the development and progression of liver disease. Additionally, congruent with the purported role of GPR109A in the regulation of inflammation, the expression of key proinflammatory cytokines was also upregulated in aged *Gpr109a^-/-^* mice compared to age-matched WT mice (Fig. S2). Therefore, lack of GPR109A expression in Kupffer cells may contribute towards the proinflammatory changes seen in liver of *Gpr109a^-/-^* mice.

Our morphometric and histopathological evaluation of livers from WT and *Gpr109a^-/-^* mice, showed that in the absence of GPR109A expression, aging induces severe hepatic steatosis even when mice were maintained on a standard laboratory chow. Further, GPR109A expression declines significantly with aging in normal mice, contributing likely to the increased accumulation of fat and alteration of lipid metabolism that is known to occur in normal aging. This is logical given that the primary function of GPR109A is metabolic-sensing [17] and it is plausible that loss of GPR109A over the course of time diminishes the metabolic-sensing capacity of mice leading to aberrant hepatocyte fat accumulation. Hepatic steatosis can occur as a consequence of imbalance between *de novo* lipogenesis and fatty acid oxidation [32]. During lipogenesis, acetyl-CoA is converted to malonyl-CoA by ACC and malonyl-CoA is then converted to palmitate by FAS [32]. These fatty acids then undergo a range of modification steps to get converted into triglycerides. Sterol regulatory element-binding protein 1c (SREBP1c) is one of the main transcription factors regulating *de novo* lipogenesis [33]. Aging significantly increased expression of these lipogenic mediators in *Gpr109a^-/-^* mice compared to WT. Hepatic fatty acid oxidation is controlled by PPARα [34]. One of the key mechanisms of lipolysis is fatty acid oxidation. For the fatty oxidation process, CPT1 is responsible for transport of fatty acids into mitochondria, where ACOX1 is first enzyme for fatty acid beta-oxidation, which catalyzes the desaturation of acyl-CoAs to 2-trans-enoyl-CoAs [35]. Livers of aged *Gpr109a^-/-^* mice displayed significantly decreased expression of PPARα, CPT1 and ACOX1 indicating attenuated fatty acid oxidation.

In adipocytes, activation of GPR109A results in inhibition of adenylate cyclase and subsequent reduction of HSL/ATGL activity, resulting in reduced hydrolysis of TG and FFA and glycerol release [36]. However, little is known about the role of GPR109A expression in *de novo* lipogenesis. In the present study we observed that with aging, *Gpr109a^-/-^* mice accumulated significant fat resulting in adipocyte hypertrophy. PPARλ and CEBPα are the main transcriptional factors regulating adipogenesis [37] and in turn, affect downstream enzymes such as ACC and SCD1 to control lipogenesis in adipose tissue. Interestingly, expression of these transcriptional factors and enzymes was significantly up regulated in *Gpr109a^-/-^* mice compared to WT mice. It is interesting to note that while, activation of GPR109A results in lipolysis, and its absence enhances *de novo* lipogenesis. Since, GPR109A expression in adipocytes reduces with aging [38], it is reasonable to speculate that loss of GPR109A with age would affect *de novo* lipogenesis.

We did not observe changes in the circulating lipids of 12-month-old *Gpr109a^-/-^* mice compared to WT mice (Fig. S1). However as mentioned earlier, mice were maintained on a standard laboratory diet and therefore were not exposed to an excessively high level of external dietary lipids. So, despite the fact that basal *de novo* lipid metabolism was affected in both liver and adipose tissue, those changes were not reflected in circulating lipid profile.

Collectively, our results provide comprehensive evidence for the expression of GPR109A in hepatocytes and its role in regulating *de novo* lipogenesis in liver and adipose tissue in aging mice. GPR109A expression and activation can be accomplished readily via simple dietary approaches such as use of a ketogenic diet or intermittent fasting, methods that increase plasma concentrations of β-hydroxybutyrate, an endogenous ligand for GPR109A. Additionally, an FDA approved therapy (Tecfidera, Biogen IDEC) in which the principal bioactive component, monomethylfumarate is an agonist of GPR109A [39] is available for potential repurposing. The beneficial effects of enhancing GPR109A-mediated signaling have been demonstrated in age-related conditions such as Parkinson’s and Alzheimer’s disease, however, the efficacy of therapeutic activation of the receptor has not been evaluated in liver, in conditions of aging or otherwise. Thus, future studies to determine efficacy of these dietary manipulations in regulating age-associated hepatic steatosis and related liver conditions are warranted.

## MATERIALS AND METHODS

### Animals

All studies were conducted in accordance with the Guide for Care and Use of Laboratory Animals prepared by the United States National Academy of Sciences (National Institutes of Health) and approved by the Institutional Animal Care and Use Committee. Male C57BL/6J mice were obtained from Jackson laboratory and the National Institute of Aging (NIH). *Gpr109a^−/−^* [10] and *Gpr109a^mRFP^* mice [13], generous gifts from Dr. Stefan Offermanns, Max-Planck-Institute for Heart and Lung Research, Germany were inbred and maintained in the animal facility of Augusta University. All animals were housed under identical conditions in a pathogen-free environment with a 12:12 h light/dark cycle and free access to standard laboratory chow and water. Food intake and body weights were recorded weekly over the period of 12 months.

### Blood and tissue collection

Portions of mouse liver were stored in formalin, RNAlater (Sigma-Aldrich, St. Louis, MO) and/or and snap frozen in liquid nitrogen for further analysis. Blood samples were collected in micro-container tubes by cardiac puncture and centrifuged to obtain serum.

### Histology

Formalin-fixed, paraffin-embedded liver and adipose tissue sections were stained using Hematoxylin and Eosin (H&E). A semi-quantitative scoring system was used to assess hepatocyte steatosis by investigators blinded to the study (0, 1-5% of total area; 1, 5 – 33% of total area; 2, 33 – 66% of total area; 3, >66% of total area [40]. Adipocyte diameter and surface area were calculated using Adiposoft- ImageJ software.

Oil red O staining of liver sections was performed as described earlier [41]. Briefly, cryosections (6-8 microns in thickness) were air-dried and fixed in buffered formalin for 10 min. Sections were washed with water, rinsed in 60% isopropanol and stained with 0.3% Oil Red O solution for 15 min. After rinsing with 60% isopropanol, sections were stained with haematoxylin for 2 min, rinsed in water and mounted using an aqueous mounting media. Photographs were taken using Zeiss Axioplan imaging microscope at 20X magnification.

### Serum biochemistry measurements

Circulating levels of lipids and liver enzymes, ALT and AST, were determined in serum samples obtained from wild type and *Gpr109a^-/-^* knockout mice using a Piccolo xPress Biochemistry Analyzer and associated lipid panel plus reagent disc.

### Liver triglyceride assay

A triglyceride assay kit was used to measure the triglyceride content in livers as per the manufacturer’s instructions (Cayman, USA). In brief, 50 mg of liver tissue was homogenized in NP40 substitute assay reagent, centrifuged and supernatant collected. In a 96-well plate, 10μl of supernatant was then mixed with 150 μl of enzyme mixture solution, incubated at room temperature for 15 min and read at 530 nm using a plate reader.

### Quantitative real-time polymerase chain reaction (qPCR)

Total RNA was extracted from mouse livers and cultured liver primary and transformed mouse and human cells using miRNeasy kit (Qiagen, USA), and its quantity and quality were assessed using a ThermoScientific 2000 nanodrop spectrophotometer (Thermo Scientific, Wilmington, DE). cDNA was prepared using iScript cDNA Synthesis Kit (Bio-Rad, Hercules, CA) and subjected to real-time PCR using gene specific primers. Primer sequences are listed in Table 1. Assays were performed in 96-well PCR plates using All-in-One™ qPCR Mix (Genecopia, USA). The reaction volume of 20 μl contained 10.0 μl SYBR green master mix (2X), 1 μl cDNA, 1 μl of each primer and 7 μl nuclease-free water. The following two-step thermal cycling profile was used (StepOnePlus Real-Time PCR, Life Technologies, Grand Island, NY): Step I (cycling): 95 °C for 5 min, 95 °C for 15 s, 60 °C for 30 s and 72 °C for 15s for 40 cycles. Step II (melting curve): 60 °C for 15 s, 60 °C 1 min and 95 °C for 30 s. The template amplification was confirmed by melting curve analyses. mRNA expression of genes was normalized to 18s expression and fold change in expression was calculated by the 2^–∆∆Ct^ method.

### Isolation of independent liver cell populations

Stellate cells, hepatocytes, endothelial cells and kupffer cells from mouse livers were isolated per our previously described method [19]. Cells were maintained at 37°C and 5% CO_2_ and qPCR analyses of GPR109A expression was carried out as described above.

### Cell culture

AML12 hepatocytes (ATCC, Manassas, VA) were cultured using 1:1 mixture of Dulbecco’s modified Eagle’s and Ham’s F12 medium (Genesee Scientific, San Diego, CA) with 0.005 mg/ml insulin, 0.005 mg/ml transferrin, 5 ng/ml selenium, 10% fetal bovine serum at 37°C with 5% CO_2_. Cells were sub-cultured (1:4–1:6) using a 0.25% (w/v) trypsin-0.53 mM EDTA solution (Genesee Scientific, San Diego, CA). HepG2 and HEPA 1–6 cell lines (ATCC, Manassas, VA) were cultured and sub-cultured similarly except in Dulbecco's Modified Eagle's Medium (DMEM) containing 10% fetal bovine serum (FBS) and 1% antibiotic–antimycotic solution at 37 C with 5% CO_2_.

### Statistical analysis

Results are presented as mean ± S.E.M for a minimum of three independent experiments. Statistical significance was defined as p< 0.05 and determined using student’s t-test (normally-distributed data). Graphs were prepared using GraphPad Prism 7 software.

## SUPPLEMENTARY MATERIAL

Supplementary Figures
