# Supplementary material for: Loss of GPR109A/HCAR2 induces aging-associated hepatic steatosis
Source: Aging (Albany NY). 2019 Jan 18;11(2):386–400. doi: 10.18632/aging.101743 (PMC6366969; doi:10.18632/aging.101743)
Supplement: Supplementary Figures [file aging-11-101743-s001.pdf]

SUPPLEMENTARY MATERIAL

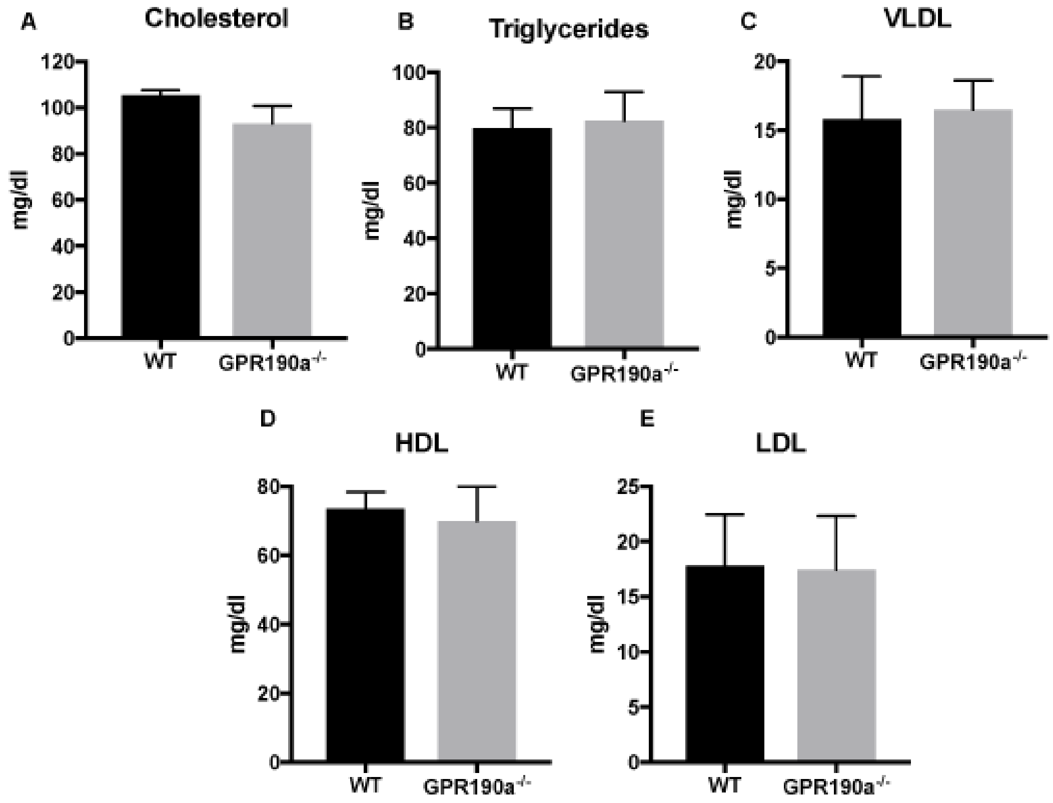

Figure S1. Increased weight gain in *Gpr109a*$^{-/-}$ mice is not associated with change in circulating lipids. **(A-E)** Serum obtained from 12-month-old WT and *Gpr109a*$^{-/-}$ mice was used for serum lipid profile analysis using piccolo lipid panel plus reagent. Serum levels of cholesterol, TG, VLDL, HDL and LDL were similar in both groups. Data is represented as mean ± S.E.M for (n=6). *p<0.05 vs. WT.

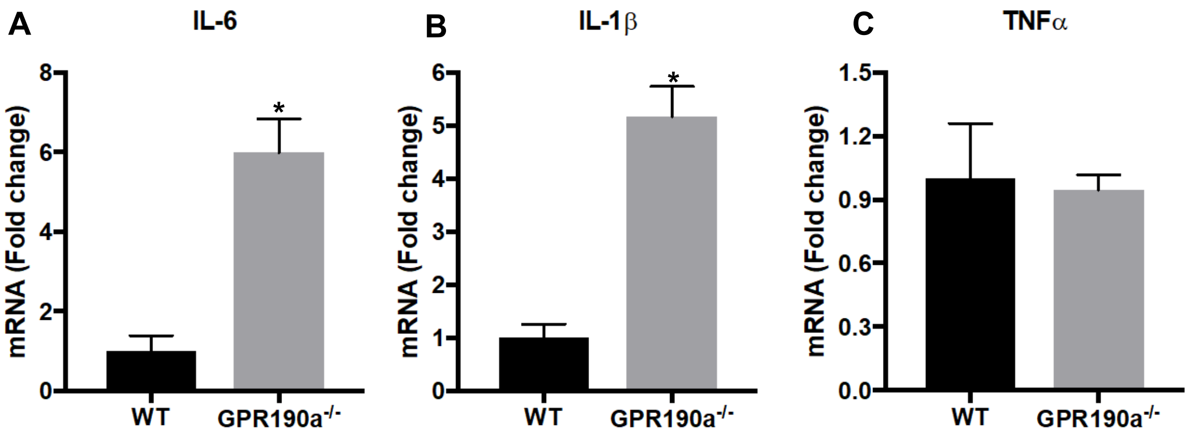

Figure S2. Loss of GPR109A increases expression of inflammatory markers in liver. **(A-C)** mRNA expression of proinflammatory cytokines was performed by qPCR assay. Data are presented as mean ± S.E.M for (n=4). *$p<0.05$ vs. WT. IL-6; Interleukin 6, IL-1β; Interleukin 1 beta, TNFα; Tumor necrosis factor alpha.
